# Developing a dashboard to meet the needs of residents in a competency-based training program: A design-based research project

**DOI:** 10.36834/cmej.69682

**Published:** 2020-12-07

**Authors:** Robert Carey, Grayson Wilson, Venkat Bandi, Debajyoti Mondal, Lynsey J. Martin, Rob Woods, Teresa Chan, Brent Thoma

**Affiliations:** 1 Department of Emergency Medicine, University of Saskatchewan, Saskatchewan, Canada; 2 Department of Computer Science, University of Saskatchewan, Saskatchewan, Canada; 3 Division of Emergency Medicine, Department of Medicine, McMaster University, Ontario, Canada; 4 McMaster program for Education Research, Innovation, and Theory (MERIT), McMaster University, Ontario, Canada

## Abstract

**Background:**

Canadian specialty programs are implementing Competence By Design, a competency-based medical education (CBME) program which requires frequent assessments of entrustable professional activities. To be used for learning, the large amount of assessment data needs to be interpreted by residents, but little work has been done to determine how visualizing and interacting with this data can be supported. Within the University of Saskatchewan emergency medicine residency program, we sought to determine how our residents’ CBME assessment data should be presented to support their learning and to develop a dashboard that meets our residents’ needs.

**Methods:**

We utilized a design-based research process to identify and address resident needs surrounding the presentation of their assessment data. Data was collected within the emergency medicine residency program at the University of Saskatchewan via four resident focus groups held over 10 months. Focus group discussions were analyzed using a grounded theory approach to identify resident needs. This guided the development of a dashboard which contained elements (data, analytics, and visualizations) that support their interpretation of the data. The identified needs are described using quotes from the focus groups as well as visualizations of the dashboard elements.

**Results:**

Resident needs were classified under three themes: (1) Provide guidance through the assessment program, (2) Present workplace-based assessment data, and (3) Present other assessment data. Seventeen dashboard elements were designed to address these needs.

**Conclusions:**

Our design-based research process identified resident needs and developed dashboard elements to meet them. This work will inform the creation and evolution of CBME assessment dashboards designed to support resident learning.

## Abstract (fr)

**Contexte:**

Les programmes canadiens de spécialité sont à implanter la compétence par conception (CPC), un programme d’éducation médicale par compétences qui nécessite des évaluations fréquentes des activités professionnelles confiables. Pour servir aux fins d’apprentissage, la grande quantité de données d’évaluation doit être interprétée par les résidents, mais peu de travaux ont été réalisés pour déterminer comment la visualisation et l’interaction avec ces données peuvent être soutenues. Dans le cadre du programme de résidence en médecine d’urgence de l’Université de Saskatchewan, nous avons cherché à déterminer comment les données d’évaluation de la CPC de nos résidents devraient être présentées pour soutenir leur apprentissage et pour développer un tableau de bord qui réponde aux besoins de nos résidents.

**Méthodologie:**

Nous avons utilisé un processus de recherche orientée par la conception pour cerner les besoins des résidents en lien avec la présentation de leurs données d’évaluation. Les données ont été recueillies au cours du programme de résidence en médecine d’urgence de l’Université de Saskatchewan grâce à quatre groupes de discussion de résidents qui se sont tenus sur une période de 10 mois. Les groupes de discussion ont été analysés en utilisant l’approche de la théorisation ancrée (Grounded Theory) pour cerner les besoins des résidents, pour guider le développement d’un tableau de bord contenant des éléments (données, analyses et visualisations) qui soutiennent leur interprétation de leurs propres données. Les besoins identifiés sont décrits à l’aide de citations des groupes de discussion ainsi que de visualisations des éléments du tableau de bord.

**Résultats:**

Les besoins des résidents ont été classés sous trois thèmes : 1. être guidés quant au programme d'évaluation, 2. présenter des données d’évaluation en milieu de travail, et 3. présenter d’autres données d’évaluation. Dix-sept éléments du tableau de bord ont été conçus pour répondre à ces besoins.

**Conclusions:**

Notre méthode de recherche orientée par conception a permis de cerner les besoins des résidents et d’élaborer les éléments d’un tableau de bord pour y répondre. Ce travail servira de base à la création et à l’évolution des tableaux de bord d’évaluation en CPC conçus pour soutenir l’apprentissage des résidents.

## Introduction

The Royal College of Physicians and Surgeons of Canada has implemented competency-based medical education (CBME) through the *Competence by Design* (CBD) program.^1^ Programmatic assessment is a core element of CBD.^2^ It requires faculty to provide frequent, low-stakes assessments of entrustable professional activities EPAs) which include both a numerical rating of resident entrustability and narrative feedback.^3^^–^^5^ Within emergency medicine (EM), 28 EPAs are mapped across four stages of training.^6^ Since CBD’s implementation, the volume of assessments has increased substantially relative to the traditional assessment program.^6^^–^^8^ As a result, the importance of effective presentation of assessment data has been characterized as critical within the early CBME reviews, commentaries and program evaluation literature.^9^^–^^13^

EPAs can be used both for the assessment *of* learning and *for* learning.^14^ Assessment for learning occurs through reflective practice and self-regulated learning.^6^^,^^15^^–^^17^ which requires a clear and accessible presentation of assessment data. Fortunately, sophisticated analytical and visualization techniques have been developed in other professional fields (e.g. business and sport) that effectively present large amounts of data.^18^ Dashboards are often used to visually display important information and allow its monitoring.^19^^,^^20^ They have been used in medical education to present learning analytics data to learners to enhance their experience.^21^^,^^22^ We sought to develop a dashboard to support resident learning using an iterative design-based research process ^23^^,^^24^ that incorporates collaboration with and feedback from information technology experts, assessment experts, data managers, and dashboard users.^11^

Within the University of Saskatchewan emergency medicine residency program, we investigated how our residents’ CBME assessment data should be presented to support their learning and developed a dashboard meeting their needs.

## Methods

To meet this objective, we employed a design-based research process^25^–^27^ and followed best practices in dashboard design^11^,^28^ including the collection and analysis of iterative feedback from our resident stakeholders. We report the results of our qualitative analysis in compliance with reporting standards for qualitative research.^29^,^30^. Our research methodology was deemed exempt from ethical review by the University of Saskatchewan Research Ethics Board (BEH ID 463).

### Settings and participants

This project was situated within the Royal College of Physicians and Surgeons of Canada EM residency program at the University of Saskatchewan between July 1, 2018 and September 30, 2019. All residents in our Royal College EM program transitioned to the EPA-based *Competence By Design* program in July of 2018. There were 14 full time residents enrolled within the program from then until June 30, 2019 and 18 enrolled from July 1-September 30, 2019.

All resident participants were asked via email by their colleagues (RC and GW) to participate in focus groups that were held in our EM Resident Library. Participation in focus groups was voluntary and food was provided at each session.

### Research team

Our research team included two residents (RC and GW), an established medical education researcher (BT), our program director (RW), our competence committee chair (LM), an external expert in medical education research and assessment (TMC), a computer science research assistant (VB), and a computer science professor (DM).

### Design based research process

The Design-Based Research methodology (11,24) that we employed aligned with the approach outlined in our previous work on competency committee dashboards.^31^ Design-based research is an “authentic, contextually aware, collaborative, theoretically focused, methodologically diverse, practical, iterative, and operation-oriented” process^11^,^25^ which aims to bridge practice and research in education through the integration of investigation and intervention.^24^^,^^25^^,^^27^ Our process consisted of the four phases of design-based research.^25^

### Phase 1. Analysis and exploration

The senior author (BT) reviewed the literature on reflective practice and self-regulated learning,^15^-^17^ learning analytics,^18^,^32^ and data visualization^11^,^20^,^23^,^32^ to generate ideas for the initial iteration of the resident dashboard. In November 2018, our resident investigators (RC and GW) facilitated simultaneous focus groups lasting 64 minutes. Data collection included field notes taken during the meeting, transcribed audio recording, and visuals drawn by the participants. The guiding questions asked of the focus group were: What assessment data does our program collect? Is this assessment data used to guide your learning? If so, how? How could this assessment data be presented to support your learning?

### Phase 2. Design and construction

The initial focus group data were transcribed and qualitatively analyzed to inform the creation of a dashboard prototype. Two authors (VB and BT) then met between two and four times monthly to discuss the results and design the prototype dashboard. Given the overlap between the needs of the competence committee^31^ and the residents, the initial dashboard was very similar for each of the groups. The first dashboard prototype was released to residents in December 2018.

### Phase 3. Evaluation and reflection

Phases 2 and 3 alternated into the next year with each of the additional three focus groups spurring the creation and evolution of dashboard elements. The subsequent three focus groups were held in March, June, and September of 2019 and lasted 48, 30, and 38 minutes, respectively. During each, the EM residents reviewed the dashboard and were asked: How are you using the dashboard? What information needs to be added? How could the assessment data be presented to better support your learning? Following each of the focus groups, the narrative data was transcribed and qualitatively analyzed to inform the development of the thematic framework and the evolution of the dashboard (Phase 2).

### Phase 4. Implementation and spread

The final phase describes the implementation and spread of the dashboard at and beyond our institution. Uptake in other contexts demonstrates the generalizability of our findings and dashboard to broader contexts. As this phase does not contribute directly to the determination of resident needs, we did not include it with our results.

### Qualitative analysis

Narrative data from the focus groups included drawings, field notes taken by the facilitators, and transcribed audio data. The data was analyzed through a constructivist grounded theory approach to identify the core needs for resident assessment data. Dashboard elements (data, analytics and visualizations) were designed to meet these needs and spurred discussion at subsequent focus groups regarding the optimal presentation of the data they included. Comments related to resident perspectives on their progress and general comments regarding CBD were excluded from the analysis.

The qualitative analysis was conducted using a constructivist grounded theory approach and constant comparative method.^33^ Following the first focus group, two authors (RC and GW) independently developed codebooks with representative quotes for each code. They then met and amalgamated their codebooks by adding, modifying, and removing codes on a consensus basis. One author (RC) compiled the codes into a preliminary framework of resident needs. Following each subsequent focus groups, the same authors coded the data and refined the thematic framework while selecting representative quotes for each need. BT reviewed all the transcripts, codes, and the framework intermittently to ensure that it was comprehensive and representative of the data. He provided additional suggestions to refine the thematic framework throughout the analysis process. He also liaised directly with the programming team (DM and VB) to prioritize updates to the resident dashboard. The resulting thematic framework was described using representative quotes as well as images of the dashboard elements mapped to each theme.

The investigators considered their own positionality and its potential impact on their data interpretation throughout the coding process. RC and GW were both emergency medicine residents within the program who regularly utilized the dashboard during most of the coding period (GW transferred to another residency program on July 1, 2019 but continued to contribute to the data analysis until the project was complete). BT is an emergency physician who was a Residency Program Committee member during the period of study. He previously served as the Program Director, CBD Lead, and Competence Committee Chair of the residency program. We acknowledge that the close involvement of the three coding investigators with the residency program was likely to impact their interpretations of the data, however, their involvement in this capacity was a pragmatic decision that allowed rapid and contextualized coding and reduced delays in the iterative dashboard design process.

Participant checks occurred in two ways. First, each of the residents were asked to review the thematic analysis and provide feedback on anything that was out of keeping with their perspective. Second, the residents were consulted throughout the dashboard development process and had the opportunity to provide feedback when the dashboard elements did not meet their needs.

### Data management and dashboard programming

All EPA assessment data for our residency program was entered by faculty into the Royal College of Physicians and Surgeons Mainport ePortfolio (Ottawa, ON). The data was then exported and uploaded to the dashboard each Monday by the emergency medicine Program Administrator. During the upload process, EPA data was reformatted and tagged with the rotation each resident was in when each EPA was completed. Contextual and non-EPA information (e.g. resident name, program start date, phase of training, rotation schedule, exam scores) was entered into the dashboard by the Program Administrator. All dashboard data was stored on a secure server in the Department of Computer Science at the University of Saskatchewan.

The dashboard was developed on a distributed web architecture with three components: a database server to securely hold the data, a web server for hosting the website, and a back end server to authenticate users and perform CRUD (create, read, update, and delete) operations.^31^ This allowed each of these parts to be updated independently, which facilitated rapid prototyping based on user feedback. This also allowed the dashboard to be easily adopted by additional programs. Dashboard visualizations are rendered in a scale and transform invariant Scalable Vector Graphics (SVG) format that make the user experience consistent across various screen sizes and orientations. Logging into the dashboard required authentication through the University of Saskatchewan’s Central Authentication Service. Access to data was restricted based on pre-assigned user roles with residents restricted to viewing only their own data. The dashboard source code was published on GitHub^34^ under an open access license to allow its rapid replication by other institutions. There are no plans to commercialize the dashboard.

## Results

Seven to 10 residents from a variety of postgraduate years participated in each of the four focus groups (Table 1). The preponderance of male residents reflected the gender balance of the residents in the residency program during the period of study (12 male and two female at the time of the first three focus groups; 12 male and five female at the time of the final focus group).

**Table 1:** Participating residents and demographics over the four focus groups held.

Focus Group Date	Gender of participants	Residency year of participants	Research phase
November 7, 2018	Male - 8 Female - 2	PGY1 - 2 PGY2 - 3 PGY3 - 3 PGY4 - 1 PGY5 - 1	Analysis and Exploration
March 13, 2019	Male - 6 Female - 1	PGY1 - 3 PGY2 - 2 PGY3 - 2 PGY4/5 - 0	Evaluation and Reflection
June 19, 2019	Male - 7 Female - 1	PGY1 - 2 PGY2 - 3 PGY3 - 3 PGY4/5 - 0	Evaluation and Reflection
Sept 11, 2019	Male - 6 Female - 1	PGY1 - 1 PGY2 - 4 PGY3 - 1 PGY4/5 - 1	Evaluation and Reflection

The qualitative analysis identified three resident needs (Table 2). Each need has been described with representative quotes and linked to their corresponding dashboard elements in Table 2. Given the limitations of tables and figures, a video was developed to provide a dynamic demonstration of the dashboard and how it addressed each resident need (Video 1).

**Table 2:** Thematic analysis of resident needs and the dashboard elements developed to address them.

Resident Needs	Dashboard Element			Quotes
1. Guidance through the assessment program				
	1.1 Calendar (Figure 1)			Yeah, so just forecasting and planning life in general, it’s good. (FG#4)
		1.1.1 Rotation Schedule (Figure 1)		The other thing I like to use it for is using it to kind of plan my life out because it has all of the dates of my rotations kind of in a convenient space. And so when I question what block I’m on in three blocks from now I don’t have to like sit and sift through an Excel file; it’s right at the top of the bar, one click away, so it’s really convenient in that respect. (FG#4)
		1.1.2 EPA acquisition percentage per rotation (Figure 1)		And then also I usually just even then view EPAs per block just to see where I’m sitting in terms of number of EPAs achieved per block just to make sure I’m trying to hit that 100+% per rotation is important to me. It’s a very logical interface. (FG#4)
		1.1.3 EPA Reference Cards (Figure 2)		I was just thinking it’d be interesting to link this to [participant’s] EPA rotation cards. So I can click on emerge and see which EPAs best for that. So that not only can–so I can see, “Okay I’m short on this EPA. Oh but I have ortho coming up and oh it’s one of the EPAs for ortho. Perfect. (FG#2)
	1.2 EPA Observation Rate (Figure 3)			Just having looked at the dashboard right now I really like the EPAs observed per week. ‘Cause it kinda gives you a better idea of where you’re at in terms of getting them done. (FG#3)
	1.3 Competence Committee Feedback (Figure 4)			Yeah. I was also wondering about the possibility – like every time we meet – like you know, you have your competence committee meeting, they give you feedback and things to work on. Are there ways on the dashboard that can correlate with the things that you need to do, so it’s just kind of like— ... Yeah a goals section. So it’s kind of a reminder for you anytime you look at your EPA, these are things that I need to work on. (FG#1)
	1.4 Tracking EPA achievement			When I look at the dashboard it gives me a bit of a sense of direction. Actually the first time I used it prior to a shift, I go open this up, I look at it, and I say, “I don’t need to pay attention to this particular EPA because it’s complete.” And I can get a good impression that – or vice versa. I need – I may have lots of EPAs in here but I can see that there still needs work to be done. It’s nice ‘cause it helps me channel my focus. (FG#2)
	1.5 Resident Checklist			I think courses it would be great if you just had a list of what was expected. And just a checklist where it moves off the list to another list once it’s been. (FG#1)
2. Present workplace-based assessment data				
	2.1 EPA Assessment and Feedback (Figure 5-6)			So for every EPA I want to know how many I’ve done and of the ones I’ve done, the proportion of each entrustment score I’m getting on it. (FG#1)
		2.1.1 Quantitative EPA Data (Figure 5-6)		We want to know, when we’re on a shift and we look quickly at our EPAs, we look at our foundations to training, we see we need 1.9 and I need six of those then I can just focus on that EPA if I have everything else. (FG#1)
			2.1.1.1 Line Chart (Figure 5-6)	I liked that you can hover over a point without having to click on it and can read everything. Or you can like just open the tab underneath and quickly see all of them. (FG#2)
			2.1.1.2 Filters (Figure 5)	Yeah I’d echo that. I think if I got to a point where I was nearing like 30 to 40 and I had that many and I’m like, “Wait but I need to have five kids,” then there’d be a time that I’d actually click through and be like, “Okay let’s limit it to just kids. Do I have five? No. Okay my last two are gonna just be kids.” (FG#2)
		2.1.2 Narrative EPA Data (Figure 6)		I would love that if each individual entry came up as a dot and you could like click on the entry below and go directly to the comments. So you get a timeline spread of how you’re progressing. Underneath you would have all the generic comments from that EPA so you could see your progress and it would be chronologic. So you could see all your progress as you go along but you could easily refer to- so let’s say you have like one down here afterwards – you’re like, ‘why am I sucking after a year?’ you could push it and go directly to it and understand why. (FG#1)
	2.3 Recent EPAs (Figure 7)			Another idea was just having a section with recent EPAs. Because right now, as we know, an EPA is completed and then we have to go through a series of clicks to find it. Whereas if we had one section where it just said your most recent EPAs, you could just go in [and see] what your feedback was. (FG#1)
	2.2 Expired EPAs (Figure 8)			It helped me identify like people if I work with them to really push them to get EPAs. (FG#3)
3. Present other assessment data				
	3.1 Exam Scores (Figure 9)			It’d be nice if we could also have like your CITE scores, your (oral exam) scores. (FG#2)
	3.3 Narrative Observations (Figure 10)			I also can easily access narrative EPA which is good. I think that those are really helpful for my development as well. (FG#4)

Legend: FG = Focus Groups with the residents in November 2018 (#1), March 2019 (#2), June 2019 (#3), and September 2019 (#4)

### 1. Provide guidance through the assessment program

In all focus groups, the residents were asked how they used the dashboard and what they used it for. Reinforcing the importance of this need, most residents indicated that they used it to obtain a sense of their overall progress throughout the residency program.

**Figure 1:**
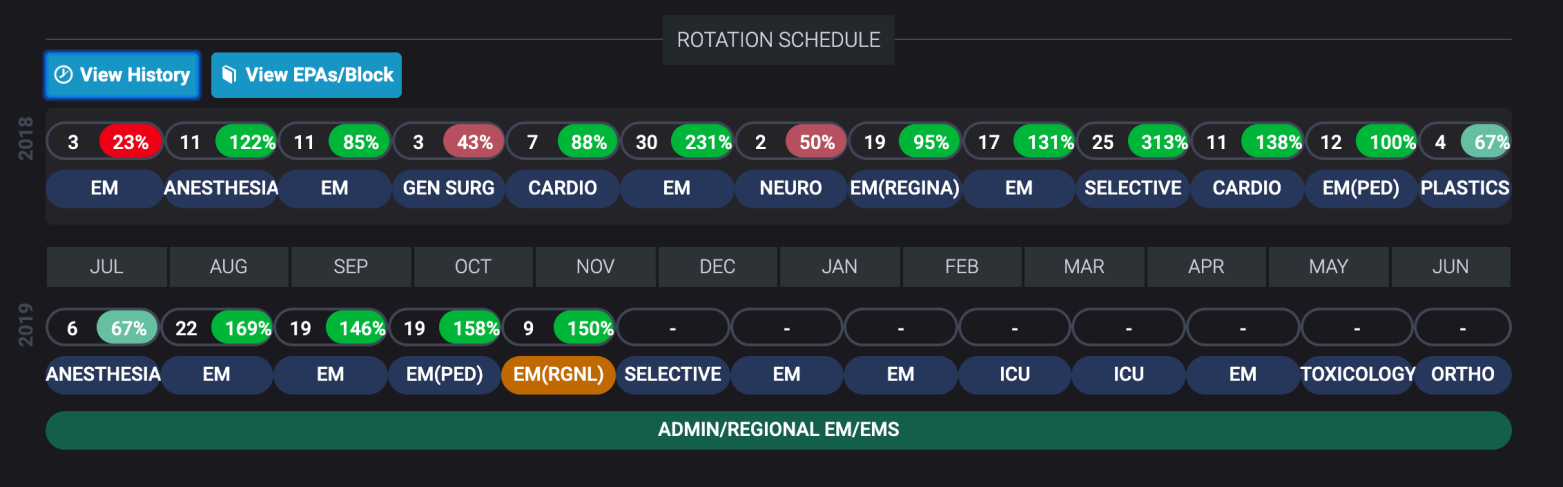
Visual representation of the rotation schedule and the number of Entrustable Professional Activity observations per block observed relative to the expected number of observations per block.

One simple element which was incorporated to provide guidance was a calendar with an incorporated rotation schedule that was always kept up to date (Figure 1). By clicking on the ‘View History’ button just above the calendar of the current year, rotations from prior years can be viewed. This provided a quick reference for which rotations have been completed each year. Additional features were also incorporated into the rotation schedule to provide the residents with further guidance on the assessment program.

The residents wanted to track the number of EPAs that they have completed during each block and whether this was an adequate amount. Selecting the ‘View EPAs/Block’ button at the top of the rotation schedule (Figure 1) produces a visualization containing the number of EPAs observed on a given four-week block along with a heat-mapped percentage outlining how that number compares to the value expected by the program. Each percentage was calculated by dividing the number of EPAs observed for the resident on that block by the number of EPAs that they should be targeting for the rotation. The target number for each rotation was based upon the number of EPAs our program’s residents have historically had observed on that rotation. In some cases, this value was modified by the program director (RW). A diverging color scale ranging from red (<25% of the expected number of EPAs) to green (>80% of the expected number of EPAs) allowed the residents to monitor their EPA acquisition relative to expectations over time.

**Figure 2:**
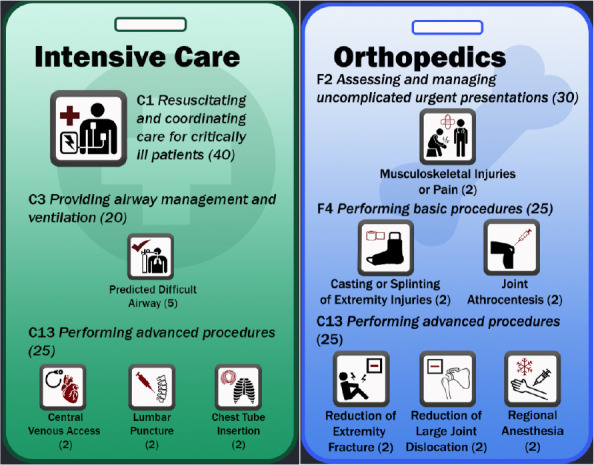
Sample of the Entrustable Professional Activity reference cards.

Program-specific reference cards^35^ were also incorporated into the rotation schedule (Figure 2). Clicking a rotation on the schedule loaded a reference card which guided the resident by indicating which EPAs should be targeted during that rotation. The design of the reference cards was based upon our program’s curriculum map and described in detail in a previous manuscript.^35^

**Figure 3:**
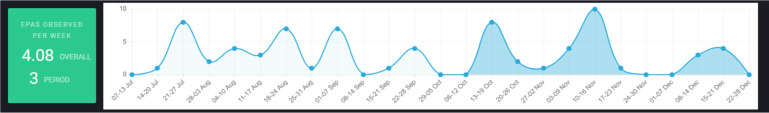
Visual representation of individual resident EPA observations per week in numerical (overall and within a selected period) and graphical (over a 6-month period) formats.

The residents were told by our program leadership that they should aim for a minimum of two EPAs per week, so they were interested in tracking the number of EPA observations they received weekly. These data were visualized through numerical acquisition metrics and a graph visualizing weekly EPA numbers during the past 6 months (Figure 3). A date filter was available for this graph to provide the number of EPAs per week received in each interval and visualize it on the graph with blue highlighting.

**Figure 4:**
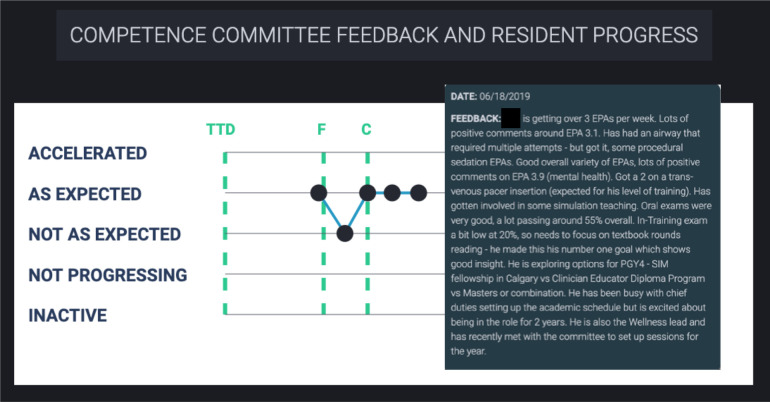
Line chart displaying competence committee decisions and an associated narrative comment.

Competence committee decisions and feedback were important to contextualize the residents’ progress. They were presented within the dashboard using a graph outlining the state of the resident’s progress (accelerated, as expected, not as expected, not progressing, or inactive) (Figure 4). Hovering over each data point displayed the competence committee’s feedback. The start of each stage of training was incorporated into the graph with vertical lines indicating the date of promotion between stages.

Within the dashboard the residents sought indications that they had demonstrated adequate competence within individual EPAs so that they could increase their focus on other EPAs. As the final determination of competence was determined by the competence committee by stage, rather than by individual EPA, it was not possible for the dashboard to display this. This feature may be incorporated into future iterations of the dashboard if the competence committee modifies their approach to competence determination.

One feature discussed at length by the residents but not incorporated into the dashboard was a “To-Do” list. The residents noted that they had numerous non-clinical responsibilities within the program (e.g. courses, poison control shifts, research, etc.) that they found difficult to keep track of. They requested that an interactive dashboard element be developed outlining these requirements and whether they had been completed within each year. This feature was not developed for this iteration as our focus was on the resident assessment features.

### 2. Present workplace-based assessment data

**Figure 5:**
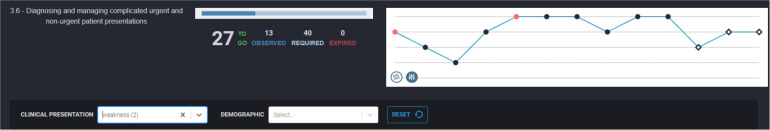
Line chart for a single Entrustable Professional Activity demonstrating the clinical presentation/demographic filter.

The residents wanted a graphical visualization of their assessments for individual EPAs. During the first focus group they suggested many ways that this could be represented graphically. The final visualization was a line chart of each EPA (Figure 5). Individual EPA assessments are presented as dots with the oldest on the left to newest on the right. Each dot is plotted on a Y-axis rating their entrustability using a 5-point entrustability score^36^,^37^ (with 5 corresponding to “I did not have to be there” and 1 corresponding to “I had to do it”). Contextual information was also incorporated including the number of EPAs that needed to be observed within the assessment program, the number that had been observed, the number that still needed to be observed, and the number of each EPA that expired (EPAs that were sent to a Faculty member by the resident but not completed).

Some EPAs must be observed for specific clinical presentations or patient age groups. Tracking these presentations and patient demographics was a challenge. We added a filter option which allowed the resident to highlight data points tagged as a specific clinical presentations or age group in red (Figure 5). This allowed residents to focus their requests for assessments on clinical presentations or patient demographics that had not previously been observed.

**Figure 6:**
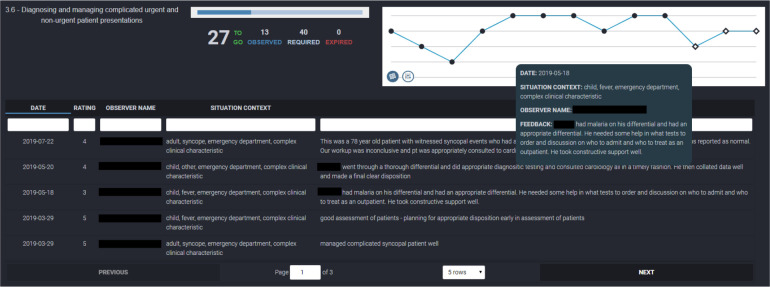
Line chart for a single Entrustable Professional Activity demonstrating two methods of displaying narrative comments.

Another challenge that the residents identified was the accessibility of the narrative comments associated with each EPA observation. We facilitated the review of these comments by developing two ways to look at narrative data within the line graph. First, hovering over the dots created a pop-up window containing the narrative comments and contextual information associated with that EPA observation (Figure 6). Second, clicking an icon in the bottom left corner of the graph presents the narrative data in a sortable, searchable tabular format (Figure 6). Both ways of viewing the narrative data met the needs identified by the residents in the focus groups with the former used for quick lookup or in-depth review.

**Figure 7:**
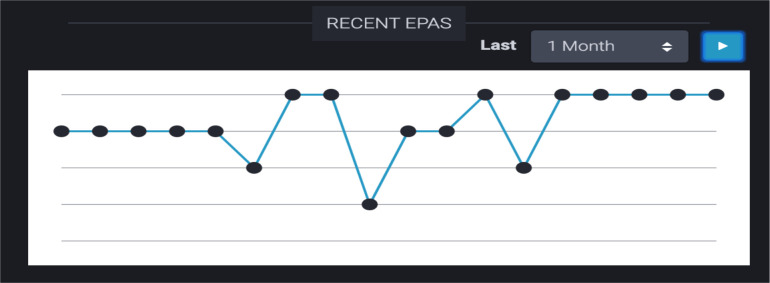
Line chart of recent Entrustable Professional Activities acquired by a single resident in a one-month time period.

A separate line chart was developed which displayed all EPAs that had been observed recently (e.g. last 10 records, last 25 records, last one month of records, last three months of records) (Figure 7). The number of EPAs presented was selected arbitrarily but felt to be useful by the residents. The time intervals were used to correspond to the length of a single four-week rotation and the timing of meetings with academic advisers (every three months). As in Figure 6, hovering over each of the dots displayed a pop-up window containing each EPA’s associated narrative comments and contextual information.

**Figure 8:**
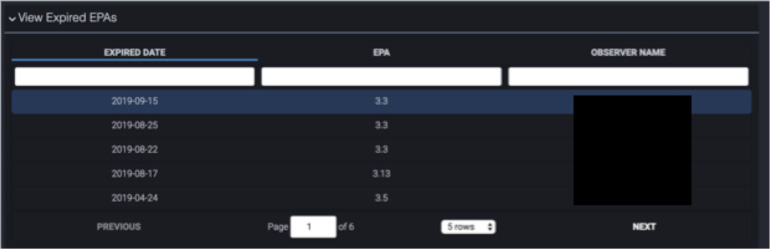
Tabular presentation of expired Entrustable Professional Activities including the date and name of the observer.

Residents often struggled to determine which preceptor(s) had been sent EPAs that they did not complete. We developed an expired EPA element to resolve this (Figure 8). Positioned at the bottom of the dashboard, it presents a table outlining the date each EPA expired, the number of the expired EPA, and the observer that it was sent to. This allowed the resident to be proactive in acquiring assessments as they could follow up with the attending physicians with outstanding EPAs.

### 3. Present other assessment data

The residents found other assessment data useful in tracking their learning and progress through residency and wanted all their assessment data available for review in a single place.

**Figure 9:**
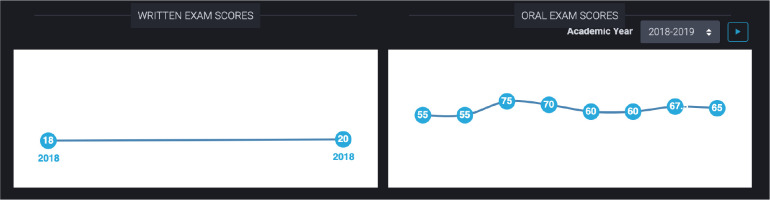
Line charts of written and oral exam scores for an individual resident.

Exam scores (Figure 9) were presented as plots over time with the numerical scores out of 100 indicated by each data point. Written exam scores were recorded as percentiles compared to other residents of the same year across Canada. All previous exam scores were contained within a single written exam graph. Oral exam scores were presented in a similar fashion but with each dot representing the score on a single oral exam station rather than the overall score for a year. Each academic year could be selected using a drop-down menu. Hovering over each oral exam data point presented the narrative comments provided by the examiner for each oral exam.

**Figure 10:**
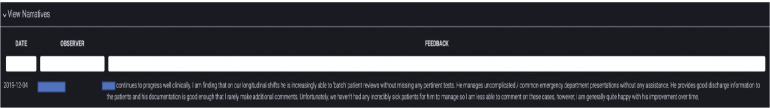
Tabular presentation of a single narrative assessment of an individual resident.

In addition to EPA observations, faculty within our program can submit general narrative assessments focused on either a resident’s overall progress or on a particular event. Neither of these assessments are associated with a specific EPA. The residents identified that they wanted access to these assessments to supplement their EPA assessments. These data were presented in a searchable and sortable tabular format (Figure 10) containing the date submitted, the name of the faculty who submitted it, and the narrative comment.

### Implementation and spread

The resident dashboard has been utilized by other local residency programs (Anesthesia, Pathology, Obstetrics and Gynecology, Internal Medicine, General Internal Medicine, and Neurosurgery) and modified for the University of Saskatchewan undergraduate medical education program. Nationally programs across Canada have expressed interest in the project. The senior author (BT) has met with representatives from the Royal College of Physicians and Surgeons, the University of Calgary, Laval University, the University of Alberta, the Elentra Consortium, and multiple national educators to discuss how it could be used by or might inform the work of other institutions with a similar goal of supporting resident learning. Resident Doctors of Canada has also requested additional information regarding the project via email. Feedback from these interactions was not incorporated into the qualitative data analysis as it was outside of the scope of the research ethics application and did not come from the perspective of resident learners.

## Discussion

Building on our previous work exploring the needs of faculty members who serve on CBD competence committees,^31^ we utilized a design-based research method to identify resident needs for data, analytics, and visualizations of their assessment data and created a dashboard to facilitate resident learning.

This paper utilized design-based research to identify and address resident needs in a competency-based medical education program. While early program evaluation efforts have identified this type of data visualization as essential,^9^^,^^10^^,^^13^ many of the studies on resident learning are largely theoretical.^12^^,^^15^^,^^16^^,^^18^ More advanced work has investigated the use of dashboards to support the reflection of radiology residents when recording their exposure to specific radiographic findings.^38^ Another study evaluated the McMaster Modular Assessment Program (McMAP) and found that EM residents valued the feedback generated by CBME and that workplace-based assessments allowed them to engage in informed self-assessment.^39^ The McMAP assessments generated improved verbal and written feedback from faculty to learners,^40^ which then became a platform from which a trainee could springboard a data-driven approach to their own self-assessment.^39^ Similarly, while the impact of the dashboard was not formally evaluated, the focus group comments suggest that it enhanced resident reflection and helped them create learning goals, thus supporting self-assessment. Such reflection is essential for resident development^16^ and thought to support self-regulated learning .^15^^,^^17^

While there are numerous overlapping elements between our competence committee and resident dashboard, it is important to note that they did identify different needs. This suggests that the use of a single dashboard for both groups without modification is not ideal.^31^ For example, the residents discussed the potential value of anonymized norm-referenced data, but the consensus was that it would foster competition within an otherwise cohesive resident group. The residents were also less focused on acquisition metrics (Figure 3) than the committee members. Conversely, residents were more focused on specific information regarding expired EPAs (Figure 8) and facilitated guidance through assessments (Figures 1-4). The expired EPAs table (Figure 8) allowed the residents to follow up with assessors. These features were not as helpful for the competence committee. Both groups had their needs addressed by the inclusion of non-workplace-based assessment data (Figures 9-10), the tracking EPA assessments over rotations (Figure 1), and the individual EPA visualization (Figures 5-6).

There were several advantages to our research approach. It provided a scholarly framework within which we were able to support the creation and evolution of the dashboard, investigate residents’ needs within a CBME program, and provide detailed visualizations to contextualize these needs. Conducting this study in a scholarly (rather than commercial) environment allowed direct access to the dashboard’s end-users while also facilitating its open-access publication for use by anyone with adequate technical expertise.^34^

### Future directions

We anticipate that resident dashboards will become an important feature of CBME assessment programs as programs increasingly seek to utilize assessment data for learning.^14^ As CBME dashboards are adopted more broadly, research will be needed to quantify the impact of their designs on resident on resident reflection, learning, and self-regulation as well as their impact on residents’ relationships with their mentors/coaches. Dashboard designers should pay attention to the potential for their own perspectives and biases to be perpetuated within the design of resident dashboards in ways that could be detrimental to learning. Having developed dashboards to address the needs of both competence committee and residents, we plan to continue our design-based research process to investigate the needs of faculty developers and program leaders who are supporting CBME assessment programs.

## Limitations

There were limitations to our project. First, the data were collected within one EM residency program at a single center in Canada which may limit its applicability to other disciplines or programs. However, the specifications of *Competence by Design* are consistent across Canadian medical specialties and its enthusiastic use by multiple non-EM training programs suggests that many of our findings are transferrable. Second, while we are confident that our thematic framework provides a robust representation of resident needs, as the residents gain more experience with CBME the dashboard elements will likely need to evolve. The involvement of residents (RC and GW) in the data collection process may have biased the results as they were active members of the program that was studied. We attempted to remediate this through the inclusion of a non-resident investigator (BT) in the qualitative analysis process, but he is also actively involved in the program in an academic role. We also note potential gender bias in our focus group participants due to the gender breakdown of our program. Notably, during development the substantial overlap between the competence committee and resident dashboards resulted in a core dashboard that served two purposes. Lastly, while there were no substantive design conflicts between the two groups, occasionally features were added to the core dashboard that were more desired by one of the groups than the other. This led to the dashboard being influenced by both the competence committee focus groups and resident focus groups.

## Conclusions

This project identified multiple needs for the presentation of assessment data to residents within CBME programs. The resulting dashboard and its supporting thematic framework should inform the development and evolution of resident dashboards at other institutions.
